# Stress and sport performance: a PNEI multidisciplinary approach

**DOI:** 10.3389/fpsyg.2024.1358771

**Published:** 2024-03-01

**Authors:** Giulia Tossici, Valentino Zurloni, Andrea Nitri

**Affiliations:** ^1^“Riccardo Massa” Department of Human Sciences for Education, University of Milano-Bicocca, Milan, Italy; ^2^Health Hub srl, Lurate Caccivio, Italy

**Keywords:** stress, sport performance, allostatic load, coping, stress management interventions

## Abstract

Stress control is essential for avoiding a state of anxiety in sport competitions, as this state may have negative effects on other psychological variables of athletes, decreasing their self-confidence and harming their attentional control. In the present contribution a distress intervention model developed from a PNEI perspective will be sketched out. Our theoretical-methodological proposal consists of the definition of an integrated protocol of psycho-biological assessment and intervention on the allostatic load and on the levels of distress/eustress detectable in the sport environment, in relation to the person’s health/well-being condition and the impact of this condition on the quality of sport performance.

This paradigm has the potential to explore both the psychological dimension of stress management and the psycho-educational and psycho-physical dimension, according to a truly integrated approach to the athlete’s health and psychophysical well-being. Its multidisciplinary nature requires close cooperation between different professional figures, such as the mental coach, psychologist, nutritionist, osteopath, and physiotherapist, as well as biologists, physicians and kinesiologists, both in planning and in implementation and monitoring at all stages. The potential impact of the model on sport performance will be deeply discussed.

## 1 Introduction

### 1.1 Stress and performance

#### 1.1.1 Stressors in sport

In the sports arena, athletes face numerous stressful situations, which are generated from different sources. In general, situations that are regarded as challenging, potentially threatening, or requiring a considerable waste of resources to deal with (coping strategies) are categorized as stressors. These may include environmental factors related to competitive performance, such as participation in major competitions, rivalry with an opponent, media attention, unsatisfactory refereeing, unfavorable weather conditions, or a decline in performance (Ntoumanis and Biddle, 1998). Aspects related to the sports organization of which the athlete is a part, such as economic insecurity, communication problems with the coach or teammates in team sports, training methodologies, a change of role, conflicts of values (Buceta, 1985), can also be a source of stress. Likewise, events that are part of an athlete’s life, such as the death or illness of a close person, or a change of residence can affect anxiety and stress levels in an athlete (Arnold and Fletcher, 2021). In addition to external factors, numerous internal factors can be a source of stress. Even if the study of personality in sports psychology is primarily focused on investigating the associations between personality, participation, and athletic achievement (Aidman and Schofield, 2004; Allen et al., 2013; Allen and Laborde, 2014; Steca et al., 2018), such association may also influence perceived stress in athletes. A variety of motivational and dispositional variables that are correlated with sports performance and success has been investigated (e.g., Steca et al., 2008; Baretta et al., 2017).

Exposure to such stressors can negatively affect an athlete’s health (Simms et al., 2020), well-being (Roberts et al., 2019) and performance (Arnold et al., 2017; Arnold and Fletcher, 2021). Conversely, some studies have shown how certain stressors, including injuries, can be associated with more positive outcomes (e.g., stress-related growth, Roy-Davis et al., 2017). The literature on this topic does not always agree in identifying stressors that negatively affect the athlete, for several reasons. The potential effects such situations may have are largely moderated by the athlete’s appraisal of stressful situations. The stress response is determined by three factors (Anderson and Williams, 1988): personality characteristics (rigidity/flexibility, locus of control, trait anxiety, achievement motivation, sensation seeking); the athlete’s personal history of coping with stressors, regarding both severe and minor events; coping resources and social support. Furthermore, most research has focused on the analysis of individual stressors (e.g., competition, organizational or personal factors) rather than exploring their cumulative effects on health (Fletcher et al., 2006). To assess the effects of negative events on performance, several studies recurred to self-report checklists (Moore et al., 2018), which can assess the frequency of a relatively small number of events (such as the loss of a loved one) while neglecting some key dimensions of stressors (such as magnitude and severity; Slavich, 2019).

#### 1.1.2 Effects of stressors in sport

Many athletes struggle to implement functional strategies to cope with the causes and consequences of stressful events, with outcomes that can have a very negative impact on their performance and health. Such difficulties in managing high levels of anxiety and stress in sport can lead to a variety of outcomes, including unsatisfactory performance, negative thought patterns, negative emotions and depressive symptoms, and injuries (Buceta, 1985). In contrast, athletes who possess a broad repertoire of coping strategies govern stressful situations more effectively and achieve optimal levels of anxiety/arousal. This has a positive impact on performance.

According to the anxiety/stress spiral model (Cox, 1998) athletes with high levels of trait anxiety manifest distress-related symptoms more frequently (Man et al., 1995). Three distinct dimensions are involved in competitive anxiety experience: cognitive anxiety, somatic anxiety and self-confidence (Hardy, 1990, 1996; Martens et al., 1990; Maynard and Cotton, 1993; Hardy et al., 2004). While an adequate level of somatic anxiety can be beneficial (an inverse U-shaped correlation with performance has been observed; Burton, 1988), increased cognitive anxiety negatively correlates with performance (Cox, 1998). In other words, improving performance requires reducing cognitive anxiety and negative thoughts, and finding the optimal level of somatic anxiety. However, it appears that the construct of sport-specific anxiety is a better predictor of performance than generalized anxiety, and that this correlation is influenced by numerous individual factors such as locus of control, self-efficacy, and sport confidence (Felsten and Wilcox, 1992). Similarly, the correlation between anxiety and performance also takes on different trends depending on the type of sport practiced. For example, it has a significant negative impact in sports that require high levels of concentration and motor coordination (Felsten and Wilcox, 1992). Individual and contact sports are associated with high levels of cognitive and somatic anxiety, while sports involving individual scores based on judges’ assessments predict high levels of cognitive anxiety (Martens et al., 1990). Self-confidence and self-efficacy are important variables related to sport performance (Robazza and Bortoli, 2007) since they increase perceived ability to emotion regulation and provides possibility for athlete to manage negative emotions more effectively (Besharat and Pourbohlool, 2011). High levels of self-confidence in athletes are associated with perceived useful ability (e.g., Martens et al., 1990; Robazza and Bortoli, 2007). It also moderates competitive anger symptoms (Hanton and Connaughton, 2002; Hanton et al., 2003), and facilitates coping resources for encountering anxiety (Jones and Hanton, 2001; Hanton and Connaughton, 2002; Robazza and Bortoli, 2007). Self-confidence before and during the match determines lower level of competitive anxiety and often correlates with better performance (Craft et al., 2003).

High stress levels can also generate burnout phenomena (Silva, 1990). Overwork during training is linked to a deterioration of immune functions, increased negative emotions and increased fatigue (Perna et al., 1998). Even regular training sessions can lead to negative consequences in case of conflicts, boredom, poor coping, or irregular work/rest patterns (Silva, 1990). Silva pointed out that repeated failure to cope with demands and discouragement related to continuous efforts that are ineffective can lead to burnout in sport, resulting in withdrawal from sport, low self-esteem, and loss of athletic identity (Silva, 1990). Furthermore, psychological stress is associated with increased levels of the stress hormone cortisol; in the case of chronic stress, athletes may be more frequently subject to injuries and illnesses caused by a lowered effectiveness of the immune system (Perna et al., 1998). By causing an excessive increase in concern about performance and the outcome of a competition, sub-optimal anxiety and the resulting increase in stress may also hinder the athlete in achieving an optimal state of flow (Kimiecik and Stein, 1992).

To cope with stressful situations in both sport and everyday life, it is necessary for athletes to master a wide repertoire of effective coping strategies to regulate suboptimal levels of anxiety (Lazarus, 2000; Schinke et al., 2012; Crocker et al., 2015; Nicholls et al., 2016a, 2016b). The scientific literature on the topic has demonstrated the effectiveness of some stress management programs in reducing anxiety and stress, with a positive effect on performance (Gould and Udry, 1994; Rumbold et al., 2020).

#### 1.1.3 Coping and performance

Performance is the measurable result of a series of activities executed by the subject. In the sport domain it represents the result of a competition and how it took place, as it can be used to assess the ability of an athlete or team. The effects of coping strategies on performance have been extensively explored by the transactional model of stress (*Cognitive-motivational-relational theory,* CMRT; Lazarus, 1991, 1999). In the case of sports, athletes assess the subjective valence (primary appraisal) of the demands (internal and/or external) of a situation. The primary appraisal would determine whether these demands are negligible or relevant. The former would not require elaborate coping strategies, nor would they impact on the athlete’s commitment, values, or goals. In contrast, when faced with demands that are deemed relevant (stressful), primary appraisal allows predictions to be made as to whether the athlete perceives the situation as an obstacle or threat (anticipated or experienced loss), or as a challenge (anticipated or experienced gain). Secondary appraisal allows one to assess the coping strategies available to cope with the situation, and how well the athlete feels he or she can govern the situation and the emotions related to it (Doron and Martinent, 2021). According to Nicholls et al. (2016a, 2016b), coping strategies would be differentiated into: *mastery coping*, i.e., strategies through which the athlete tries to suppress the stressor by attempting to take control over the stressful situation (e.g., problem-focused coping and task-oriented coping); *internal regulation coping*, through which the athlete tries to manage internal responses to stress (e.g., acceptance, emotion-focused coping); *goal withdrawal coping*, which refers to situations in which the athlete abandons all attempts to achieve his or her goal (e.g., disengagement-oriented coping). These strategies have a major impact on sport outcomes, improving or worsening performance, facilitating, or hindering the achievement of sport goals, and modifying emotional experiences (Nicholls and Polman, 2007; Crocker et al., 2015; Nicholls et al., 2016a, 2016b). Specifically, problem-focused coping strategies are found to correlate positively with performance and positive emotions, whereas disengagement-oriented and emotion-focused coping strategies have a negative association with performance and a positive association with negative emotions (Nicholls and Polman, 2007; Crocker et al., 2015; Nicholls et al., 2016a, 2016b).

According to the CMRT model, stress appraisal, coping behavior and emotions are dynamic psychological adaptation processes that enable us to adapt to and cope with the physical, psychological, and social changes we undergo throughout life (Lazarus, 1991, 1999; Nicolas et al., 2017). Stress would be the result of the interaction between individuals (cognitive appraisal and coping) and environmental factors. Consequently, the stress response also includes closely related physiological, cognitive, emotional, and behavioral components. Even though the CMRT assumes a strong interdependence between the psychological constructs at play (Lazarus, 1991, 1999), most studies have adopted approaches focusing on one or at most two constructs (Martinent and Nicolas, 2017; Doron and Martinent, 2021). More recently, a few studies have explored the relationship between stress appraisal, emotions, coping and sport activity outcomes (well-being or performance satisfaction), by structural equation modeling (Nicholls et al., 2012, 2014, 2016a,b; Britton et al., 2019; Thompson et al., 2020), but results are controversial. The relationships between these processes, and how their individual fluctuations are related to individual performance fluctuations require more process-centered and systemic approaches (Lazarus, 1991, 1999, 2000).

### 1.2 Stress management interventions

#### 1.2.1 Stress assessment

Although the impact of stressors on well-being and health is widely recognized in the literature, empirical studies on the subject are scarce (Slavich and Shields, 2018). To date, there are no assessment tools that systematically evaluate the impact of stressor exposure (Slavich, 2019). Despite the diversification of stressors, the possibility that they emerge at different times and domains of life, and the interaction with socio-psychological characteristics, stress has been considered as a unitary construct (Epel et al., 2018). This results in a simplified view of the effects of stressor exposure (Epel et al., 2018).

The *Stress and Adversity Inventory* (STRAIN; Slavich and Shields, 2018) is often used to investigate the association between exposure to stressors and psychological, biological and well-being aspects. Its application in various fields has shown a strong correlation between stressors and depressive symptoms (e.g., Pegg et al., 2019), anxiety disorders (e.g., Slavich et al., 2019) and other physiological disorders (e.g., respiratory tract infections; Cazassa et al., 2020).

Most studies focusing on the effects of stress in sport have only considered certain types of stressors (e.g., related to competition, organization, or personal factors), rather than exploring the combined and cumulative effects of stressors on health (Fletcher et al., 2006). To assess their effects on athletes, such studies have made use of self-report checklists relating to traumatic or particularly significant events (e.g., Moore et al., 2018). Despite the advantage of being easily administered due to their brevity, checklists are limited to assessing the frequency of an extremely limited number of stressors, neglecting several dimensions that render the complexity of the phenomenon (Slavich, 2019).

To date, only one study has used STRAIN to assess correlations between stressors, mental health, and well-being in sports (McLoughlin et al., 2021). The results showed a correlation between chronic disorders and exposure to stressors with symptoms of anxiety, depression, and lower psychological well-being in elite athletes. Follow-up interviews revealed that cumulative exposure to stressors negatively impacts mental health and well-being because it: facilitates the sedimentation of non-adaptive coping strategies; increases susceptibility to future stressful experiences; and creates difficulties in interpersonal relationships (McLoughlin et al., 2021). However, the study did not assess the effect of sport-specific stressors, nor their impact on performance, and was limited to a sample of elite athletes.

McLoughlin et al. (2022) recently proposed an adaptation of the STRAIN to stress in sport (Sport SAM), analysing its usability, acceptability, validity, and test–retest reliability. The scale, which was administered to a sample of 395 sportsmen and women, showed a correlation with depressive and anxious symptoms as well as mental and physical disorders. Furthermore, it showed that the correlation between the severity of stress events (sports and non-sports) and health is mediated by trait stress appraisals (McLoughlin et al., 2022). In contrast to the self-report checklists used in previous studies, the Sport SAM considers the combined and cumulative effects of sporting and non-sporting stressful events and examines the effects of aspects related to the athlete’s health, well-being and performance rather than focusing on only one of these aspects (Moore et al., 2018; Fletcher, 2019). However, some limitations of the study require further investigation. Firstly, the cross-sectional nature of the study design limits the possibility of determining causal relationships between the variables involved. Secondly, self-report measurements may be subject to cognitive bias and social desirability. Finally, the smallness of the sample and the average age (23 years) limit the generalisability of the results. Future research needs to enrich the promising results obtained through the Sport SAM through the implementation of longitudinal studies, the application of objective measures that are not susceptible to self-report biases (assessment tools that evaluate the effects of stressors on physiological markers, such as immune response, or trait stress appraisals, such as cardiovascular reactivity; Hase et al., 2019), and through the design of intervention protocols capable of lowering the effects of stress on health and performance.

In recent years, much attention is being paid to the analysis and evaluation of increasingly specific biomarkers, detectable by blood, urine and salivary tests and samples. In the current state of the art, the most closely monitored values concern dehydration (through sodium and creatinine analysis) to closely monitor weight and electrolyte changes in athletes, muscular tissue status, endocrine changes and cardiovascular changes through the evaluation and analysis of biomarkers such as cortisol, testosterone, DHEA and IGF-1 (Lee et al., 2017). Biomarkers relating to the state and risk of injuries, recovery after physical exertion and inflammation are further indicators worthy of attention.

#### 1.2.2 Stress management

Starting from this theoretical framework, the importance of broadening the range of research-interventions to better understand which approaches are most functional for managing stress in the context of sports performance is widely recognized (Jones and Hardy, 1990; Anshel, 2005; Thomas et al., 2008; Rumbold et al., 2020). The studies to date that have proposed stress management interventions applied to sport have mostly been guided by the transactional stress process model. However, the declinations they have followed are different and have focused on one of several constructs at play, including: reduction of stressors, change in cognitive appraisals, reduction of negative emotions and increase in positive ones, and facilitation of effective behavioral coping strategies. The debate on the effectiveness of such treatments is still open. Optimizing an intervention should consider all components of the stress process, in their interactions and dynamic interdependence (Rumbold et al., 2020).

Among the most frequently used stress management treatment programs, *Stress Inoculation Training* (SIT; Meichenbaum and Deffenbacher, 1988; Meichenbaum, 2008) has achieved some positive outcomes in the field of sport (Long, 1984; Whitmarsh and Alderman, 1993; Holm et al., 1996). SIT involves three phases: conceptualisation, skill acquisition, and application. The skill acquisition phase, central to the program, consists of training cognitive strategies and makes use of techniques such as relaxation, controlled breathing, attention diversion and imagery, and positive self-talk (Kerr and Leith, 1993). Applications of SIT have proven effective in decreasing anxiety states, improving performance in studies (college athletes; Holm et al., 1996), and improving positive self-statements (Long, 1984). However, research has found no significant differences in performance compared to the control group.

The *Cognitive-Affective Stress Management Training* (SMT; Crocker et al., 1998) focuses on controlling emotional arousal through relaxation and cognitive techniques. Similarly to SIT, It consists of three phases, but adds emotional induction techniques in participants: after imagining stressful situations that increase anxiety levels, athletes are asked to adopt coping strategies (including self-talk and relaxation) to reduce anxiety (Crocker et al., 1988). Although the athletes reported fewer negative thoughts in response to videotaped stressors and improved service reception in volleyball training applications compared to the control group, no differences were found with the control group with respect to competitive state or trait anxiety (Crocker et al., 1998).

The *Cognitive-Behavioural Stress Management Intervention* (BCSM, Perna et al., 1998) has also been used in sport with promising results. The central component of the program is cognitive restructuring, a technique that involves identifying negative and dysfunctional thought patterns and learning to replace them with positive and self-affirming thoughts (Beck, 1984). Although with numerous variations in its applications, BCSM consists of a psycho-educational component (informing athletes about stress and its effects) and the use of cognitive (cognitive restructuring) and behavioral (muscle relaxation) techniques. In an application with college athletes, participants showed a decrease in negative emotions, fatigue, and stress hormone cortisol levels (Perna et al., 1998). Another study showed a decrease in anxiety and an increase in academic performance in the experimental group compared to the control group (Holm et al., 1996). However, these studies did not consider treatment outcomes on sports performance, and are limited to a population of student athletes, which invalidates their generalisability.

Systematic reviews that have attempted to summarize the outcomes of intervention programs in sport are few and dated. From the data collected from the 23 interventions on athletes included in their review, Greenspan and Feltz (1989) concluded that the most effective strategies to improve athletes’ performance are relaxation-based strategies and cognitive restructuring programs. Martin et al. (2005) included 15 studies in their review, the majority of which focused on multi-modal cognitive-behavioral-based programs built on individual athletes. The authors report positive effects on performance in most of the studies considered. However, these reviews only considered interventions focused exclusively on improving performance, neglecting both intervention programs aimed at optimizing stress management in athletes and psychosocially oriented treatments aimed at improving the athlete’s well-being in general (Miller and Kerr, 2002; Rumbold et al., 2020). Extending their review to include these criteria, Rumbold et al. (2020) considered 64 studies, which can be categorized into three different intervention types: *cognitive interventions*, in which the treatment consists of cognitive-behavioral therapy, coping, goal setting, hypnosis, imagery, rational-emotive therapy, and self-talk; *multimodal interventions*, which involve various combinations of: arousal control, attentional training, centering, cognitive control, cognitive and somatic relaxation training, concentration, COPE therapy, energizing, goal setting, hypnosis, imagery, meditation, motivation, pre-performance routines, positive thinking, self-talk, stress inoculation training, team building, thought stopping, and visuomotor behavior rehearsal; *alternative interventions*, which consist of anger awareness, applied relaxation, biofeedback, music interventions, personal goal management, and progressive relaxation training. From the scholars’ analysis, multimodal treatments appear to be the most effective. However, the wide diversification in terms of intervention techniques adopted makes it difficult to understand which of these techniques combine best to produce effective outcomes. Furthermore, while interventions aimed at stress management have been shown to be effective in improving athletes’ stress management in competitive sports, interventions aimed at also improving performance have shown fewer convincing outcomes. Finally, many studies have some methodological limitations that invalidate the generalisability of the results (i.e., small sample, no control group, and no manipulation check).

In general, the limitations of research in stress management to improve sports performance suggest some guidelines that need to be considered. First, individual differences in anxiety levels, performance and coping strategies must be considered within more personalized pathways (e.g., with accurate cognitive/somatic anxiety assessments). Such differences may affect the outcomes of treatments focused on single components of the stress process, which may be effective for some athletes and less effective for others. There is a need to move toward multimodal and multidimensional treatments that are guided by clear and well-defined theoretical models.

## 2 The PNEI proposal to stress management in sport

### 2.1 Stress from a PNEI perspective

In today’s scientific landscape, a paradigm that seems to have much to offer to meet the above-mentioned needs, namely (1) the personalisation of treatments; (2) the multidimensionality of diagnostic processes and intervention models; (3) the integration of psychological and somatic components; is that of **Psychoneuroendocrinoimmunology** (PNEI; Ader, 1981), a discipline that has gained increasing consensus and credibility in the scientific community in recent decades.

Within this paradigm, which is inspired by the research on stress conducted in the middle of the last century by the Hungarian-born, naturalized Canadian physician and scientist Hans Selye, the knowledge acquired from endocrinology, immunology and neuroscience has gradually converged during the 20th century. This convergence gives PNEI a transversality that characterizes its approach to both research and treatment, aimed at studying the functioning of the organism in an integrated manner and the bidirectional relationships between psychological and biological systems.

Stress is one of the mechanisms that best lends itself to this integrated consideration and therefore constitutes one of the privileged fields of study of PNEI. The updated edition of the Handbook of Psychoneuroendocrinoimmunology (Bottaccioli and Bottaccioli, 2016) defines it as the fundamental way in which the organism, in its entirety, adapts to the physical and social environment. It is not one mechanism among others, which is activated only in the face of internal or external challenges that belong to the register of exceptionality, but rather a normal condition of life and a common phenomenon experienced in everyday life: not the exception to the rule, but the very rule of life (including sport activities).

Stress processes are the privileged mechanism through which the individual perceives, processes, and responds to the challenges of the environment, both physical and psychological. Such challenges are global since they invest the entire complexity of the human experience, as its activators cover a wide and transversal range of biological, psychological, and social factors. The stress response unfolds according to the same pattern (the stages and curve of stress discovered by Selye), but in the face of a wide range of different stressors: physical environmental factors (e.g., heat, noise, etc.), endogenous factors of physiological nature (e.g., reduced blood pressure, lowered immune defenses, etc.) or extraordinary and all-encompassing factors (e.g., infections, hemorrhages, etc.), pathological conditions of organic origin (e.g., chronic inflammation, serious pathologies, etc.), but also emotional and cognitive factors, thus linked to the individual’s affective, relational and social life. Based on this unspecificity of the stress response, (Selye, 1974, 1977) theorized the existence of a ‘general adaptation syndrome’ (GAS), a vehicle for normal adaptation to the environment inside and outside the organism.

Today, research in this field has confirmed and deepened these insights, and PNEI, in particular, has contributed to innovating our knowledge in three respects: (1) the nature and functioning of the psychobiological appraisals that govern the stress response (with the elaboration of the notion of **allostasis**); (2) the centrality of **interoception** mechanisms which lies beneath stress regulation processes; (3) the role of the environment as a moderator and activator of the stress response and its impacts on the organism (lifestyle research related to **epigenetics**). From the intersection of these three aspects has emerged an innovative and powerful concept of stress from both a diagnostic and therapeutic point of view, capable of firmly embedding the psychological components of stress (appraisals and coping styles, as they were already explored, among others, by the Lazarus’ transactional model) in the physical and biological realm of the body.

#### 2.1.1 Allostasis and psychobiological regulation

Considering this new set of knowledge, the stress system is indeed revealed to be an integral part of the ever active and largely unconscious interoceptive network (brain’s *intrinsic* activity) that issues predictions about the body, tests the resulting simulations against sensory input from the body, and updates your brain’s model of the body in the world (Seth, 2015; Barrett, 2017). It is a complex and sophisticated predictive system of adaptation to the environment, which processes predictions about the body’s *internal milieu*, correlating it to the continuous variations and changes in the external environment, to ensure that the organism adapts as well as possible (*fitness*) to its context. And this also by way of derogation, if necessary and within a certain margin of tolerance, from the homeostatic parameters of bodily physiology. This is an important extension of the traditional physiological conception, more closely anchored to the idea of homeostasis as the restoration of equilibrium prior to the stress condition, which opens to a more elastic and flexible functioning with respect to environmental variations, capable of renegotiating extra-homeostatic conditions of adaptation named as **allostasis** (Sterling and Eyer, 1988).

The predictive and dynamic nature of allostatic mechanisms requires a more complex neurobiological direction of stress mechanisms than that of purely homeostatic systems, calling into play the brain as a whole, with the involvement of vast cortical and subcortical neural networks of control and management including, in particular, the Salience Network and the Default Mode Network (Bottaccioli and Bottaccioli, 2016; Barrett, 2017; Minelli, 2020), in functional communication with deep subcortical limbic centers (especially amygdala and hypothalamus). In the PNEI model, the mind is thus at the center of stress processes, as the governing pole of appraisals and allostatic evaluations that regulate the search for a predictive and dynamic balance with the environment. An aspect that, as we shall see, also has relevant implications on the side of stress-related treatments and therapeutic interventions, which are also applicable to the sport domain.

#### 2.1.2 Distress and allostatic load

Allostasis represents a ‘biological’ possibility for the organism, an option that can be exercised under certain conditions and within certain limits. On the one hand, it is a valuable resource for the individual since it allows an adaptation that is no longer merely reactive, but predictive and dynamic to the environment and more flexible than pure homeostatic functioning. On the other hand, by deviating from internal homeostasis, it always entails a physiological ‘cost’ for the organism called ‘allostatic load’. This load takes the form of an increase in psychobiological arousal levels and states of hypervigilance and generalized psychobiological tension in the organism.

From a PNEI perspective, therefore, three different cases are distinguished in relation to stress:

- **eustress**: activation of the stress system in which the organism overcomes the stressor(s) without exceeding permanently its homeostatic parameters. Eustress represents a positive and life-sustaining condition in which the organism does not pay any particular physiological cost at the level of its biological systems. The stress response mobilizes the energetical resources of the organism only for a limited period of time, after which the initial condition, prior to the stressful activation, is restored.
- **distress**: activation of the stress system in which the stressor(s) persists over time, and tends to chronicize the stress condition. In order to cope with this prolonged activation, the organism must deviate from its homeostasis and negotiate a different balance with the environment. This implies to pay necessarily a physiological cost of adaptation (the *allostatic load*).
- **burn-out**: a pathological condition of depletion/exhaustion of the body’s adaptive resources, brought about by the progressive accumulation of allostatic load that, beyond a certain threshold and under certain conditions, comes to render the individual unable to tolerate the stress levels of his or her environment. Burn-out, therefore, is only the last “station” of distress, the end point of a condition that gets progressively out of control; a veritable syndrome that requires medical and psychological interventions.

According to this distinction, it is therefore important to emphasize that distress does not represent a pathological condition *per se* but a possibility inherent in the degree of freedom that allostasis offers to our biological constitution. However, the accumulation of these deviations over time, with the related hyperactivation of the stress axes (mainly the HPA axis), may lead to chronic stress, to pathologize the organism’s state of health and favor the onset of numerous diseases, remain as a risk.

An increase in distress levels is closely associated with a significant increase in cortisol levels. If perpetuated over time (in a chronic sense), it leads to a progressive weakening of the athlete’s immune system and subsequent fatigability, chronic stress, and health problems (Palacios Le Blé et al., 2015). It is therefore important to monitor the levels of distress associated with different allostatic loads to prevent them from exceeding the body’s tolerance threshold.

But how and according to which methodology is such detection possible? Conventional biological approaches in stress management generally focus on the detection of specific parameters that are particularly significant for the stress condition. The focus is on individual biomarkers and countermeasures are triggered when these parameters reach levels of clinical significance, considered as conventional cut-points. Although these biomarkers are indeed linked to the stress reaction, several recent reviews have shown that this methodology shows little predictive capacity with respect to the onset of burn-out or pathological distress conditions (i.e., Danhof-Pont et al., 2011). The limitation would thus be represented not by the considered indicators in themselves, but by their separate and isolated evaluation. This approach is unable to account for the non-linear effects on physiological systems that stress exerts on the organism. Chronic stress, in fact, entails a series of impacts that accumulate over time, depending on the prolonged exposure to stressful situations and to the changes in the internal and external body’s conditions. To make the stress condition objective from a biological point of view, therefore, a broader and more comprehensive evaluation should be put in place. It is a question of constructing an integrated and multi-systemic index that is oriented toward defining the overall picture of the dysfunctions and imbalances that occur at the expense of the main physiological systems that regulate stress. As Minelli and De Bellis (2014) note, such an index, which for the following we will refer to as the **aggregate index of allostatic load,** incorporates information relating to a multiplicity of physiological systems that are involved in a functionally interconnected manner in allostatic processes; for this reason it is able to more fully reflect the cumulative effects of allostatic load on our organism (Juster et al., 2010). This is true in all stressful conditions, including the sport activity. At a professional level, in fact, sport is a privileged allostatic exercise and concerns athletes very closely: it shifts the individual’s psycho-biological limits and tolerance thresholds further and further through constant physical and mental training. Depending on the goals that the athlete intends to achieve, the prolonged and intense exercise that he or she undergoes daily determines significant deviations from the basal activity of his or her body, with modifications to both body and mind. Knowing the opportunities and risks of allostatic loading, learning how to manage it and contain the inevitable wear and tear effects, is therefore particularly appropriate for those individuals who, like sports athletes, subject their bodies to significant and constant physical and mental stressors.

#### 2.1.3 Epigenetics and lifestyles

The third contribution that PNEI offers to stress management models is the important focus on the relationship between lifestyle and eustress/distress. In the light of the most recent scientific findings on epigenetics, in fact, it becomes much clearer how, how much and through which biological mechanisms the environment comes to influence the development of the organism and the setting of the stress system itself. Many steps forward have been taken in the direction of a more complete understanding of the complex relationship between genes and environment, so much so that today it is no longer possible to consider the genome as a steering center that gives instructions to the organism (as the fathers of molecular genetics, Crick and Watson, proposed to consider it), but rather as an adaptive device that responds to environmental demands by regulating gene expression (Bottaccioli and Bottaccioli, 2023). Epigenetic research has revealed the existence of a kind of genomic plasticity that brings reversible and irreversible changes to the organism (epigenetic signatures) which, in some cases, can even become trans-generational hereditary traits.

Thanks to epigenetic mechanisms, therefore, the organism modifies itself throughout its existence, in a continuous and constant exchange with the environment and in particular with certain particularly influential factors, including (a) polluting and toxic factors (‘endocrine disruptors’), (b) the diet which, depending on the substances contained in the food, can have inflammatory or anti-inflammatory effects (e.g., excessively refined fats or sugars activate the transcription factor NF-kB, promoter of genes involved in the production of inflammatory molecules; resveratrol, curcumin, butyrate and other short-chain fatty acids work in the opposite direction); (c) physical exercise, which has proven positive effects and reduces the body’s inflammation levels; (d) emotional stress, which can deeply affect the functioning of the stress system itself.

Research in this field has allowed us to recognize the influence of all these factors on the physiology of the stress system, switching phenomena of physical and psychosocial nature, as well as our lifestyle habits, into stable changes in the organism. This awareness is remarkable in professional sports, where individuals are subject to high emotional and psychological stress loads, and where nutrition and exercise are central dimensions.

### 2.2 Potential developments of the PNEI approach in sport

In the light of these considerations, integrating this knowledge into the stress management models currently in the sport domain would be worthwhile. A PNEI model should be applied in terms of diagnostic tools, prevention interventions and treatment models for athletes both in relation to how they perceive and manage stress, and to promote an improvement in their performance under the banner of sustainability and health. Such integration opens the field to highly personalized work, since everyone is a ‘measure of themselves’ in stress, especially when the psychological dynamics of allostatic appraisals, individual habits and lifestyles, and the richness of the person’s socio-emotional experience are considered. The PNEI paradigm, in this sense, has a strong individualizing vocation and enables the development of assessment settings and treatment models characterized by much more personalized pathways.

Any intervention that wants to affect distress, in fact, must work on the individual stress management modes that people deploy to cope with life’s challenges. Such strategies are structured over time through allostatic appraisals and translated into consolidated stress management models and styles which are linked, on the one hand, to *recurring psychobiological patterns* (the cascade of bodily phenomena that accompanies the stress reaction); on the other hand, to the *coping strategies* adopted with a certain frequency. These allostatic, predictive patterns of adaptation to the environment are built up over time and become part of the largely unconscious baggage of individuals, often in the form of behavioral, cognitive, and emotional automatisms that guide personal and professional practices, not always in a functional manner. To become the object of treatment, therefore, these mechanisms require specific work to emerge and raise awareness. A distress intervention aimed at addressing these cores must focus on personal ‘self-awareness’, to favor the perception of experienced distress levels, the identification of consolidated stress management patterns and related coping strategies, as well as the quality of one’s lifestyle.

The main areas of intervention on which a distress intervention model developed from a PNEI perspective should primarily work, through the lever of self-awareness are, in our opinion, four:

1. *Interoceptive body listening*: to stimulate athletes to become more aware of the interoceptive aspects of their own psycho-biological functioning, especially with regard to the activation of stress axes;
2. *Knowledge of one’s own stress management styles,* i.e., the automatisms, coping strategies and recurring defense mechanisms that are associated with allostatic appraisals;
3. *Knowledge and reflection on lifestyles* (promoting reflection on eating habits, exercise and other variables, both physical and psychosocial), that are central to individual allostatic load from an epigenetic perspective;
4. *Activation of the* person’s *support network*, i.e., all those internal and external factors that can predispose or conversely mitigate the stress reaction (among which social support plays a key role)

The work on these dimensions, in line with the theoretical-clinical paradigm of the PNEI, is carried out from a multidimensional perspective, with the elaboration and application of a mix of diagnostic (i.e., assessment) and intervention tools that explore both the psychological dimension of stress management and the psycho-educational and psycho-physical dimension, according to a truly integrated approach to the athlete’s health and psychophysical well-being.

### 2.3 An integrated multidisciplinary approach

Based on the aspects highlighted in the preceding paragraphs, our theoretical-methodological proposal consists of the definition of an integrated protocol of psycho-biological assessment and intervention on the allostatic load and on the levels of distress/eustress detectable in the sport environment, in relation to the person’s health/well-being condition and the impact of this condition on the quality of sport performance.

This paradigm has the potential to integrate aspects of replicability with customisation requirements that are linked to the context in which the athlete operates and to her/his individual needs and specificities.

Lastly, its multidisciplinary nature requires close cooperation between different professional figures, such as psychologist (mainly, health psychologist, and sport psychologist with experience in mental coaching), nutritionist, osteopath, and physiotherapist, as well as biologists, physicians and kinesiologists, both in planning and in implementation and monitoring at all stages.

The intervention protocol, mainly addressed to elite athletes, is in its planning phase. The following are to be considered as the main drivers for its implementation.

#### 2.3.1 Methodology of intervention

The protocol consists of three phases: assessment, treatment, and monitoring.

- The **assessment** consists of profiling the athlete, aiming at reconstructing the global picture of his or her psychophysical condition, through the drawing up of a medical record containing the main information concerning the athlete’s psycho-emotional, physical, and nutritional spheres and lifestyle habits.
- The **treatment** consists of an intervention program that includes actions aimed at working on both the psychological aspects related to stress management and the psycho-educational components linked to lifestyle habits, as these are salient aspects of the athlete’s mental and physical health and well-being.
- **Monitoring** consists of the quali-quantitative assessment, during the treatment, at its end, and in the follow-up phase, of the athlete’s progress, which will be evaluated and followed up, with a view to improving and/or maintaining an optimal state of health, as well as with a view to performance and injury prevention.

##### 2.3.1.1 Assessment

The protocol starts with taking charge of the athlete and drawing up a medical record. The athlete is jointly assessed by an interdisciplinary team composed of health and mental wellbeing professionals (health psychologist, and sport psychologist with experience in mental coaching), health and physical and bodily wellbeing professionals (doctor, physiotherapist, osteopath, kinesiologist, nutritionist, etc.), laboratory experts to support the clinical analyses and biological assessments carried out, assisted by the staff that usually follows the athlete (coach, athletic trainer, etc.).

Psychologist draws up a profile of the player, assessing the following aspects:

- subjectively perceived stress load and detectable levels of psychological well-being/illness;
- the ability to listen and decode stress-related interoceptive processes and the cascade of psychobiological phenomena associated with the allostatic response;
- habitual stress management patterns and related coping strategies adopted in one’s own experience;
- established lifestyles and awareness of their impacts on stress response and levels of psychophysical well-being;
- the quality of the perceived social support network (relationship with the coach/athletic trainer/staff, relationship with the team, any perceived pressures in family and community contexts).

This evaluation requires the adoption of quali-quantitative analysis and survey tools, using batteries of reference tests/questionnaires (e.g., Strain and Sport SAM), interviews, self-reports, etc.

In addition to the usual medical assessments of the athlete’s general health condition and physical performance, the assessment protocol includes a specific activity to assess the allostatic load and stress levels detectable in the body. To this end, with the support of specialized biologists and laboratory technicians, a battery of tests and laboratory analyses is carried out, with the detection of the main salivary and blood biomarkers of stress, such as cortisol, DHEA, testosterone, oxytocin, and melatonin (administered according to the seasonal training and performance load). The detection of biomarkers is fundamental in our methodological proposal for the investigation of the main biological systems, such as neuroendocrine, cardiovascular, metabolic, immune-inflammatory and redox state. In line with PNEI approach, these indicators will not be considered in a single and unrelated manner, but rather through an integrated assessment aimed at determining the aggregate allostatic load index (Juster et al., 2010; Minelli and De Bellis, 2014), strongly indicative of the athlete’s instant stressful conditions.

To have an overall and global picture of the psychophysical condition and lifestyle, in addition to the psychological and biological assessment, the athlete is also assessed from three other points of view:

- by the nutritionist biologist, in relation to one’s eating habits and preferences, possible intolerances, allergies and sensitivities: objective measurement using BIA (bioimpedance analysis, for detecting lean mass, fat mass, water percentage and fat distribution, as well as calculation of basal metabolism). Depending on the anamnesis, further investigations may be recommended for the detection of any paraphysiological (such as dysbiosis) or pathological conditions.
- by a physiotherapist, orthopaedist, and osteopath to detect any dysfunctions and functional deficits, so as to understand the propensity to risk musculoskeletal injuries. This joint evaluation will be fundamental for the identification of postural disorders and for the detection and identification of possible painful conditions, limiting performance and/or sources of distress. The kinesiologist will then proceed with the integration of postural assessment and kinematic analysis of the main movements involved, such as analysis and measurement of the ROM (range of motion) and measurement of strength tests with a dynamometer;
- by consultants specialized in the assessment of sleep quality and sleep hygiene through specific dedicated questionnaires, to make the necessary improvements and corrections (e.g., ESS - Epworth Sleepiness scale, Johns, 1991; PSQI - Pittsburgh Sleep Quality Index, Buysse et al., 1989; SWAI - Sleep/Wake Activity Inventory, Rosenthal et al., 1993). The close connections between adequate quality of sleep and athletic performance are well highlighted by recent scientific literature.

From the integration of these evaluations and based on threshold values preliminarily identified by the research team, the population of athletes will be divided into 3 groups:

**Optimum profile (green):** the athlete appears to be in excellent psycho-physical condition and perfectly fit. No criticalities or imbalances emerge in relation to the global assessment of the psychophysical condition and lifestyles.

**Average profile (yellow)**: the athlete appears to be in good psycho-physical condition and moderately fit. Minor imbalances emerge in relation to the global assessment of the psychophysical condition and lifestyles.

**Critical profile (red)**: the athlete appears to be in poor psycho-physical condition and low performance. Extensive and widespread deficits emerge in relation to the global assessment of the psychophysical condition and lifestyles.

##### 2.3.1.2 Treatment

The treatment involves the application of a series of methods that work on the different aspects assessed during the assessment (psychological, physical, biological, stress management, lifestyle, etc.) to promote a general improvement in the athlete’s condition of life, health, and psychophysical well-being. A non-exhaustive list of tools that can be implemented within the treatment protocol includes:

- Breathing and body relaxation techniques to de-stress and reduce anxiety and tension levels (e.g., diaphragmatic breathing, autogenic training, biofeedback, meditation, etc.);
- listening and interoceptive awareness techniques to improve the athlete’s individual knowledge and sensitivity to his or her body and the cascade of psycho-biological phenomena related to the stress response (e.g., body scan, mindfulness techniques, PNEI-Med method, etc.);
- techniques for restructuring coping strategies in its different declinations (emotional, cognitive, behavioral, and social support coping) to promote the adoption of more functional and adaptive coping styles (e.g., mental imagery, stress balancing, self-talk, metacognitive and reflective tools, etc.)
- psycho-educational training for improving lifestyles oriented in particular on the dimensions of nutrition and sleep (e.g., lifestyle diary and its monitoring protocol; nutrition re-education interventions, sleep hygiene practices, etc.);
- treatments to improve the body’s general physical, inflammatory and musculoskeletal condition (physiotherapy and osteopathic treatments, acupuncture).

Based on the assessment and psychobiological profiling process, the team has at its disposal a wide range of qualitative and quantitative data useful for the strategic planning of the pathway to support the athlete’s psychophysical condition. In relation to the results of the profiling:

**Intervention 1 - Optimal profile**: periodic ongoing monitoring should be scheduled (depending on the specifics of the sport practiced and seasonality) of the biological readings related to the individual’s allostatic load, to verify any deviations from the optimal condition detected. Treatment will focus primarily on interventions of a preventive nature, with the application of stress management techniques, physiotherapy and osteopathic maintenance sessions, possible meditation and breathing techniques as needed and depending on the workload.

**Intervention 2 - Medium profile**: a specific treatment protocol will be drawn up to work on the most relevant aspects of imbalance that emerged during the assessment of the athlete’s psychophysical condition. It is fundamental to foresee a periodic ongoing monitoring of the progress made, with ongoing evaluations for the eventual reconsideration and reshaping of the treatment plan. In areas where no imbalances emerge, periodic monitoring of the biological readings linked to the individual’s allostatic load (depending on the specifics of the sport practiced and seasonality) is likewise planned.

**Intervention 3 - Critical profile**: a specific treatment protocol will be drawn up to work on the critical aspects that emerged during the assessment of the athlete’s psychophysical condition. Priority is given to the recovery of the psychophysical and metabolic condition and the prevention of injuries and relapses through a highly specific and customized approach. Compared to the type 2 intervention, therefore, a more intensive and transversal work plan is envisaged, aimed at comprehensively addressing the distress condition and insisting on all salient areas of psychophysical health and well-being. The treatment pathway will be accompanied by more frequent and wide-ranging ongoing monitoring of the progress made, with assessments for possible reconsideration and remodeling of the treatment plan. Periodic ongoing monitoring of biological readings related to the individual’s allostatic load (depending on the specifics of the sport practiced and seasonality) is also planned.

##### 2.3.1.3 Monitoring

During the treatment phase, at regular intervals or on the occasion of particular events (e.g., traumatisms, significant reduction in performance, increase in perceived levels of distress and malaise, illnesses and/or systemic pathologies) and depending on the periodisation of the training and performance load, the biomarker measurements carried out during the assessment phase will be replicated, in order to objectivise the trend of the subject’s psycho-biological condition. Continuous monitoring allows a direct comparison with previous conditions and the possibility of anticipating and preventing certain conditions that could become dysfunctional or limiting for the athlete.

At the end of the treatment, a psychological re-test phase is envisaged through the administration of the assessment tools already used in the profiling phase (e.g., STRAIN and Sport SAM), aimed at assessing the results of the treatment also from a psychological point of view (e.g., stress management, coping strategies and psychological well-being) for the overall assessment of the pathway and the possible planning of follow-up moments.

During the different phases of the path, the continuous sharing of data and information also with the team itself (teammates, coaches, trainers, managers, etc.) is crucial to encourage personal and common objectives and for full sharing of operational models, in favor of a peaceful environment and mutual trust. The athlete, once he has shared the path with the whole team, will find it increasingly easier to discuss his perceived state of stress and will be more compliant in choosing the most suitable treatment, with clear responsiveness in the medium-long term.

## 3 Conclusion

In this article, we have developed a theoretical proposal for the integration of a psycho-biological assessment protocol and a related intervention plan, which is inspired by the appraisal and stress management methodologies of Psychoneuroendocrinoimmunology.

This integration is necessary and desirable to strengthen certain aspects of stress management and performance empowerment with respect to the key dimensions identified in the specialist and reference literature in the field of sport.

In particular, the integration with the PNEI-inspired methodologies make it possible to define a solid and valid diagnostic and intervention model on a theoretical, methodological, and scientific level, which is therefore easily replicable and adaptable in different sports contexts (group and individual), at different professional levels, with respect to the many types of activities practiced, and in relation to the specific psychophysical conditions of each athlete. This replicability is supported and favored by the multidimensional and multidisciplinary nature of the PNEI paradigm, which strives to combine, in the same model, scientific knowledge and evidence from a wide range of medical, psychological, health care and physical and bodily well-being disciplines. The integration of psychological and somatic components is, moreover, one of the main valuable aspects of the PNEI paradigm’s explanatory model of stress functioning; an aspect that we also wished to apply in a sports context.

This declination has taken into strong consideration the syndromic nature of stress, which refers to a multi-componential constellation of clues and symptoms belonging to a plurality of dimensions that concern both the more physical-biological aspects of the organism and performance evaluation, and the more properly psychological, social and relational aspects. This makes the condition of stress extremely pervasive in relation to the person’s life. High levels of allostatic load and distress, in fact, can have a very relevant and all-round impact on the health and well-being of athletes. For this reason, the protocol also emphasizes lifestyle components, which can profoundly influence the development of the organism and the setting of the stress system itself, in line with the current knowledge of epigenetics.

Finally, this constellation is also shown to have a strong individual variability: it does not manifest itself in the same way for everyone and in all sports, it can also shift significantly over time, depending on the different living conditions of the person and their context. It is therefore essential that stress management protocols, in addition to being rigorous and scientifically reliable, as well as replicable, should also be able to modulate and adapt to the specific characteristics of the type of sport, the type of context, the individual uniqueness of the athlete, and the factors linked to the contingency of the specific moment/phase. Our proposal, in this sense, provides for a strong customisation and flexibility of the diagnostic, treatment and monitoring processes, to provide a detailed and global picture of the athlete’s physical and psychological condition and its possible fluctuations over time.

In the light of these considerations, it is possible to hypothesize a methodology of approach in a PNEI key that responds to the need to manage the allostatic load and the assessment of athletes’ psycho-biological distress levels, through specific, measurable, and integrated interventions, as personalized as possible. This approach, in addition to the already envisaged psycho-physical benefits in the short and medium term, could bring about an improvement in the overall management of the athlete, with respect to his or her psycho-physical health and well-being, performance and quality of life in general.

## Author contributions

GT: Conceptualization, Methodology, Writing – original draft. VZ: Conceptualization, Methodology, Writing – original draft, Writing – review & editing. AN: Conceptualization, Writing – original draft.
